# Stepped Care Internet-Delivered vs Face-to-Face Cognitive-Behavior Therapy for Pediatric Obsessive-Compulsive Disorder: A Trial Protocol for a Randomized Noninferiority Trial

**DOI:** 10.1001/jamanetworkopen.2019.13810

**Published:** 2019-10-23

**Authors:** Kristina Aspvall, Erik Andersson, Fabian Lenhard, Karin Melin, Lisa Norlin, Lena Wallin, Maria Silverberg-Mörse, Inna Feldman, Matteo Bottai, David Mataix-Cols, Eva Serlachius

**Affiliations:** 1 Centre for Psychiatry Research, Department of Clinical Neuroscience, Karolinska Institutet; 2 Stockholm Health Care Services, Stockholm County Council, Stockholm, Sweden; 3 Division of Psychology, Department of Clinical Neuroscience, Karolinska Institutet, Stockholm, Sweden; 4 Institute of Neuroscience and Physiology, the Sahlgrenska Academy, University of Gothenburg, Gothenburg, Sweden; 5 Department of Child and Adolescent Psychiatry, Child and Adolescent Psychiatry Specialized Unit, Sahlgrenska University Hospital, Gothenburg, Sweden; 6 Department of Women's and Children's Health, Uppsala Universitet, Uppsala, Sweden; 7 Institute of Environmental Medicine, Biostatistics, Karolinska Institutet, Stockholm, Sweden

## Abstract

**Importance:**

Internet-delivered cognitive behavior therapy is an effective treatment for children and adolescents with obsessive-compulsive disorder and has the potential to markedly increase access to treatment for patients while being cost-effective for health care organizations.

**Objective:**

To investigate whether internet-delivered cognitive behavior therapy implemented within a stepped care model is noninferior to, and cost-effective compared with, the gold standard of face-to-face cognitive behavior therapy for pediatric obsessive-compulsive disorder.

**Design, Setting, and Participants:**

Multicenter, single-blind, randomized clinical noninferiority trial implemented at 2 specialist pediatric obsessive-compulsive disorder clinics in Stockholm and Gothenburg, Sweden. Participants are 152 children and adolescents aged 7 to 17 years with obsessive compulsive disorder, recruited through the 2 clinics and online self-referral. Patients will be randomized 1:1 to the stepped care intervention or face-to-face therapy. Blind evaluations will be conducted after treatment and at 3-month and 6-month follow-ups. At the 6-month follow-up (primary end point), noninferiority will be tested and resource use will be compared between the 2 treatment groups. Data will be analyzed according to intention-to-treat principles.

**Intervention:**

Patients randomized to stepped care will first receive internet-delivered cognitive behavior therapy for 16 weeks; patients who are classified as nonresponders 3 months after treatment completion will receive additional face-to-face therapy. The control group will receive 16 weeks of face-to-face cognitive behavior therapy immediately following randomization and nonresponders at the 3-month follow-up will, as in the stepped care group, receive additional face-to-face therapy.

**Main Outcomes and Measures:**

Noninferiority is defined as a 4-point difference on the primary outcome measure (Children’s Yale-Brown Obsessive Compulsive Scale).

**Discussion:**

Recruitment started October 6, 2017, and was completed May 24, 2019. Results from the primary end point will be available by May 2020. The naturalistic follow-ups (1, 2, and 5 years after the end of treatment) will continue to 2025. There are no interim analyses planned or stopping rules for the trial.

**Trial Registration:**

ClinicalTrials.gov identifier: NCT03263546

## Key Points

**Question:**

Is internet-delivered cognitive behavior therapy implemented within a stepped care model noninferior to, and cost-effective compared with, face-to-face cognitive behavior therapy for pediatric obsessive-compulsive disorder?

**Findings:**

This protocol describes a multicenter randomized clinical noninferiority trial conducted in a health care setting. Participants are 152 children and adolescents aged 7 to 17 years.

**Meaning:**

This trial will add to the current knowledge base by specifically evaluating a stepped care approach to the treatment of pediatric obsessive-compulsive disorder in which patients are first offered internet-delivered cognitive behavior therapy as a low-intensity intervention, reserving higher-intensity treatments such as face-to-face therapy for those who do not benefit from the first step.

## Teaser

This protocol describes a randomized clinical noninferiority trial to determine whether cognitive behavioral therapy delivered online as part of a stepped care model is noninferior to, and cost-effective compared with, face-to-face cognitive behavioral therapy for pediatric obsessive-compulsive disorder (OCD).

## Introduction

Cognitive behavior therapy (CBT) is regarded as the gold-standard treatment for pediatric obsessive-compulsive disorder (OCD)^1,2^ and has consistently shown large effect sizes compared with both wait-list (*g* = 1.53) and various active comparators (*g* = 0.93)^3^ as well as sustained long-term effects.^4^ Furthermore, CBT is an effective augmentation treatment for patients showing suboptimal response to initial courses of both serotonin reuptake inhibitors^5^ and CBT.^6^ Unfortunately, suitably trained CBT therapists specializing in OCD are scarce and typically concentrated in large urban areas, and it is estimated that many patients do not have access to CBT.^7,8,9,10^ One way to increase the availability of CBT is to remotely deliver it online without the need to attend in-clinic sessions with a therapist.^11^ Our research group has developed a therapist-guided internet-delivered CBT (ICBT) program for youth with OCD called *BIP OCD*. The treatment is primarily self-guided, with assistance from parents or legal guardians, and supported by a designated therapist who communicates with both the child and the parent through the entire treatment. The BIP OCD program has to date been evaluated in 2 open pilot studies in both children^12^ and adolescents^13^ that showed high acceptability and safety and promising results. A subsequent randomized clinical trial including 67 patients demonstrated superiority and cost-effectiveness of BIP OCD against a wait-list control.^14,15^ Interestingly, the effects of ICBT were slightly weaker than expected but continued beyond the primary end point, with patients experiencing additional symptom improvement at the 3-month follow-up. This delayed improvement phenomenon has also been observed in other ICBT trials.^16,17^ As participants in these trials were primarily self-referred^12,13,14^ and may therefore constitute a selected group of individuals (eg, highly educated, more motivated), the generalizability of these findings to regular clinic-referred patients is currently unclear.

Before ICBT for pediatric OCD can be recommended for implementation in routine clinical care, it is essential to evaluate whether this approach is comparable or noninferior and cost-effective compared with the gold standard of face-to-face CBT. Even if ICBT were less efficacious than face-to-face CBT, it might still have a meaningful place in the health system, as its potential cost-effectiveness could outweigh its loss of efficacy. In that scenario, ICBT might be used in a stepped care model in which patients are first offered ICBT as a low-intensity intervention, reserving higher-intensity treatments that require specialist input (eg, face-to-face CBT) for more complex patients or those who do not benefit sufficiently from ICBT.^18^ Stepped care approaches are heralded as the ideal model of psychiatric service delivery^1^ but have rarely been evaluated.

We describe the trial protocol of a multicenter, single-blind, randomized clinical noninferiority trial aimed to investigate whether therapist-guided ICBT implemented within a stepped care model is noninferior to, and cost-effective compared with, the gold standard of face-to-face CBT for pediatric OCD. The main hypothesis is that the stepped care ICBT approach will be noninferior to gold-standard treatment (face-to-face CBT) in reducing clinician-rated OCD symptoms and will also be associated with lower resource use. As a secondary aim, the trial will explore whether the outcomes of ICBT differ between clinic-referred and self-referred patients.

## Methods

### Study Design

The study is a 2-site randomized clinical noninferiority trial for children and adolescents with OCD. Participants randomized to stepped care ICBT first receive ICBT for 16 weeks; patients classed as nonresponders at the 3-month follow-up are offered a course of face-to-face CBT for up to 12 weeks. Participants randomized to the gold standard treatment receive face-to-face CBT for 16 weeks; as in the stepped care group, patients classed as nonresponders at the 3-month follow-up are offered additional sessions of face-to-face CBT for up to 12 weeks. The full trial protocol is available in the Supplement.

The primary end point is 6 months after the end of the first course of treatment (6-month follow-up). Long-term effects will be investigated 1, 2, and 5 years after treatment completion. The trial design is graphically summarized in the Figure.

**Figure.**
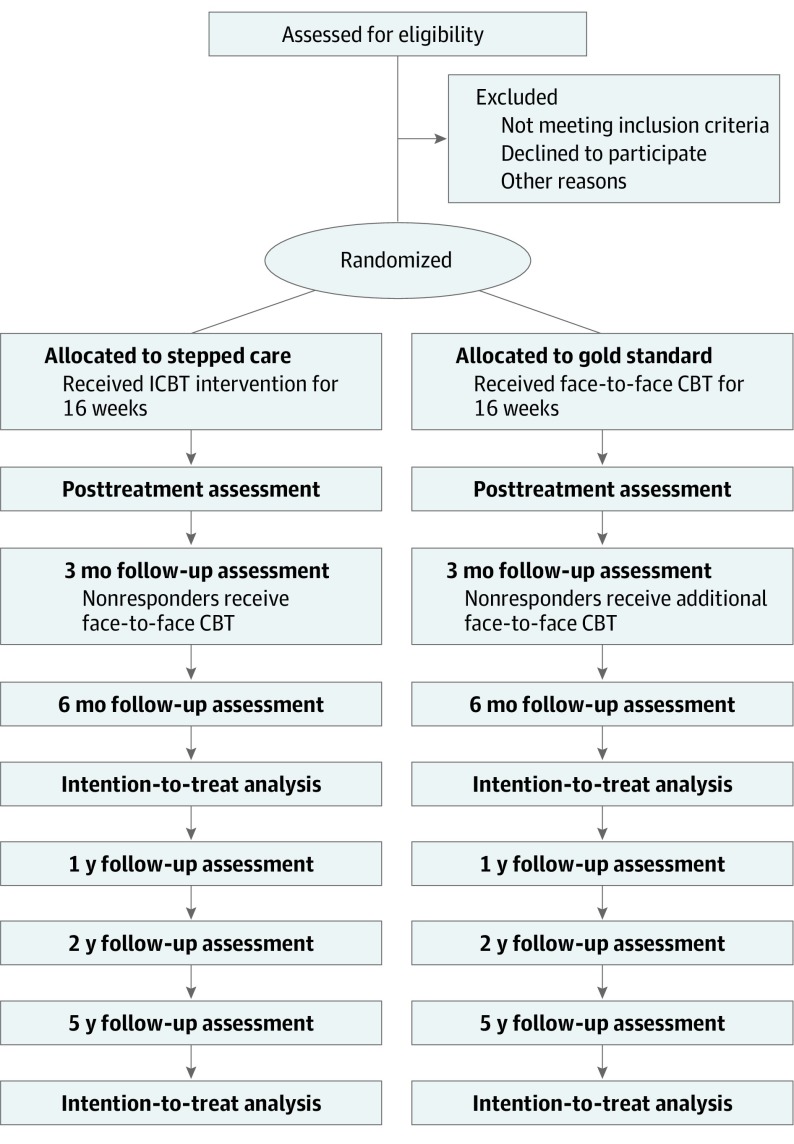
CONSORT Flowchart Abbreviations: CBT, cognitive behavior therapy; ICBT, internet-delivered CBT.

The study was approved by the Regional Ethical Review Board in Stockholm, Sweden, and preregistered at ClinicalTrials.gov. The participants and their parents received verbal and written information about the study, including an informed consent form and an age-adapted information brochure specifically designed for the children and adolescents. The written consent procedure was witnessed by the clinician who provided the verbal information about the study. This trial protocol follows the Standard Protocol Items: Recommendations for Interventional Trials (SPIRIT) reporting guideline for clinical trials.^19^

### Study Sites

Study participants are assessed and treated at 1 of 2 specialist pediatric OCD clinics, located in Stockholm (Child and Adolescent Psychiatry Research Center) and Gothenburg (Sahlgrenska University Hospital), Sweden. Stockholm and Gothenburg are Sweden’s largest cities and have similar socioeconomic landscapes.^20^ The 2 clinics receive referrals from Child and Adolescent Mental Health Services within their respective regions but also accept referrals from other parts of Sweden.

### Participants

Consecutive patients referred to the 2 specialist clinics are assessed for eligibility. Participants can also find information about the study online and self-refer via a dedicated website. Eligible participants are offered a face-to-face appointment with a clinician for a full psychiatric assessment, including the administration of the Mini International Neuropsychiatric Interview for Children and Adolescents (MINI-KID),^21^ the Children’s Yale-Brown Obsessive Compulsive Scale (CY-BOCS),^22^ the Clinical Global Impression–Severity scale (CGI-S),^23^ and the Children’s Global Assessment Scale (CGAS).^24^ If suitable for the study, both the children or adolescents and their parents sign informed consent to participate during this visit. Participants receiving psychotropic medication are requested to continue using stable doses until the primary end point (6-month follow-up).

Inclusion criteria are as follows: primary diagnosis of OCD according to the *Diagnostic and Statistical Manual of Mental Disorders* (Fifth Edition)^25^; total score of 16 or greater on the CY-BOCS^22^; age between 7 and 17 years; ability to read and write Swedish; and daily access to a computer with an internet connection. Exclusion criteria are changes in psychotropic medication within the last 6 weeks prior to baseline assessment; comorbid diagnosis of organic brain disorders, global learning disabilities, autism spectrum disorder, bipolar disorder, or severe eating disorder; suicidal ideation; being housebound or in need of intensive or inpatient treatment; having completed a course of cognitive behavior therapy for OCD within the last 12 months (defined as ≥5 sessions including exposure and response prevention); and ongoing psychological treatment for OCD or an anxiety disorder.

### Randomization and Allocation Concealment

The allocation sequence is generated by an independent external party, the Karolinska Trial Alliance (KTA). The research team and the therapists who enroll the participants are not involved in the randomization or allocation process. Participants are randomized to either stepped care ICBT or gold-standard CBT through an online automated system for clinical trials. The allocation ratio is 1:1. Randomization is stratified by source of referral (clinician-referred or self-referred) and participant’s age (children [7-12 years] or adolescents [13-17 years]).

### Quality Control

This trial follows Good Clinical Practice (GCP) standards. All quality and safety aspects are regularly monitored by KTA (ie, case-by-case monitoring of informed consent, inclusion and exclusion criteria, source data quality, and adverse events).

### Blinding

Assessments of OCD symptom severity are conducted by a team of independent evaluators blind to treatment allocation at 3 different points: posttreatment, 3-month follow-up, and 6-month follow-up. To ensure blinding integrity, participants receive explicit instructions not to disclose which treatment they have received. After completing the assessment, the blind raters guess the participant’s group allocation and record whether the family has inadvertently revealed their group allocation.^26^ If participants inadvertently disclose the treatment allocation during the assessment, another blind assessor will rerate the participant’s CY-BOCS score based on the recording from the interview. We expect that the blind raters’ guesses on allocation will not be better than chance. The participants, therapists, and the project manager are not blind to treatment allocation. The 1-year, 2-year, and 5-year follow-ups are naturalistic (unblinded).

### Assessment Points

The assessments occur at predetermined points after randomization and are independent of the treatment progress. The primary end point is at 6-month follow-up. All clinician measures and self- and parent-reported questionnaires are administered at baseline, after treatment, and at 3-month, 6-month, and 1-year follow-ups. Self- and parent-administered measures of OCD symptom severity are also administered weekly during the first 16 weeks of treatment. Additional clinician follow-up assessments are conducted after 2 and 5 years via telephone. Changes in psychotropic medication and additional psychological treatment are monitored and documented throughout the study period.

### Planned Intervention

#### Internet-Delivered CBT

The BIP OCD intervention is a 16-week therapist-guided online self-help program consisting of 14 modules provided through a secure online site with double authentication.^12,13,14^ There are 2 age-adapted versions of the intervention, one for children aged 7 to 12 years and one for adolescents aged 13 to 17 years. The content of the intervention is presented in a varied and engaging manner, with reading material, films, animations, illustrations, and exercises adapted for the 2 age groups. The main treatment components are education, exposure with response prevention (ERP), and relapse prevention. An overview of the treatment content is presented in Table 1. Parents have separate accounts and receive parallel online guidance, including in-depth knowledge about OCD, the rationale for CBT and ERP, strategies to reduce family accommodation, and ways to incorporate positive reinforcement. During the treatment, families have regular contact with a dedicated therapist with expertise in treating patients with OCD. The therapist contact is provided through written messages in the online platform or, when necessary, telephone calls. The therapist responds to all activity on the platform and sends reminders to families in case of inactivity. After treatment completion, families continue to have access to all online material for a year (without therapist support). For an overview of BIP OCD, see https://vimeo.com/355965105/b3d5d1c439.^27^

**Table 1.** Overview of the Internet-Delivered CBT Treatment Modules

Module	Child or Adolescent	Parent
Education		
1	What is OCD?	OCD and explanation of the OCD circle
2	How to get rid of OCD	Rationale for CBT and treatment goals
3	What is exposure?	What is exposure and response prevention?
Exposure with response prevention		
4	Testing exposure	Parent strategies during exposure
5	Continue with exposure	More about exposure
6	When OCD affects the family	Reducing family accommodation
7	More exposure	Motivation and parent strategies
8	Deal with the obsessions	Think about obsessions during exposure
9	New steps with exposure	Evaluate the treatment
10	Using reexposure techniques	Using reexposure techniques
11	Perform a more difficult exposure	Perform a more difficult exposure
12	Reduce avoidance behaviors	Reduce avoidance behaviors
13	Exposure and treatment summary	Exposure and treatment summary
Relapse prevention		
14	Plan for the future	Plan for the future

Abbreviations: CBT, cognitive behavior therapy; OCD, obsessive-compulsive disorder.

#### Face-to-Face CBT

Participants in the gold-standard group receive face-to-face CBT based on validated protocols,^28,29^ including up to 14 sessions with a dedicated therapist delivered over 16 weeks (allowing for missed appointments due to illness or holidays). Parents are encouraged to participate in all the therapy sessions, but their attendance varies according to participant’s age and individual needs.

The treatment begins with 2 sessions of education about OCD and the treatment rationale. The main focus of sessions 3 to 12 is ERP and includes therapist-led exposure exercises. Between these sessions, participants are encouraged to practice ERP tasks on a daily basis. Sessions 13 to 14 include relapse prevention strategies and planning to reduce remaining OCD symptoms. Sessions are typically 1 hour long and conducted at the clinic, but can be longer or conducted outside the clinic or in the participant’s home, according to individual needs. All sessions are audiotaped and a random 20% of the sessions will be selected to ensure therapists’ adherence to the treatment protocol.

#### Nonresponders

Participants in either group not meeting strict criteria for clinical response^30^ at the 3-month follow-up are offered up to 12 sessions of face-to-face CBT for OCD.^6^ This additional treatment is delivered between the 3-month and 6-month follow-ups. The content of the treatment is identical to the face-to-face CBT treatment, with the main focus on therapist-guided ERP.^28,29^ Individual adaptations are made to ensure optimal treatment dosage, including longer sessions or home visits as appropriate.

#### Therapists

The same therapists treat patients in both groups, and the families have a dedicated therapist throughout the entire treatment. Typically, the assigned therapist is the same clinician who did the baseline clinician assessment. Therapists are clinical psychologists or social workers who specialize in CBT, either experienced in delivery of CBT for pediatric OCD or recently qualified but with training and supervision in the use of the therapy manuals. All therapists have a training session on the treatment manuals and study procedures with the project coordinator prior to starting to support patients. They are closely supervised and monitored by the project coordinator, with weekly meetings throughout the trial, and can also receive on-demand supervision with 12 hours’ notice.

### Outcome Measures

#### Primary Outcome Measure

The primary outcome measure is the participant’s total score on the CY-BOCS,^22^ a semistructured clinician-administered interview for assessment of OCD symptom severity in pediatric OCD. The total score is the sum of 10 items; total scores range from 0 to 40, with higher scores indicating greater severity. The CY-BOCS has a high internal consistency and excellent interrater reliability.^22^ The CY-BOCS is administered at all assessment points. All assessors in the trial receive extensive training in using the CY-BOCS and also practice and rate cases together during the trial on a continuous basis.

#### Secondary Outcome Measures

An overview of the measurements, points for completion, and informant is shown in Table 2. The clinician-rated measures are administered during scheduled assessments at the clinic, and the child- and parent-reported measures are administered online.

**Table 2.** List of Measures and Assessment Points

Measure	Assessment Point						
	Baseline	During Treatment	After Treatment	Follow-up			
				3-mo	6-mo	1-y	2- and 5-y
Clinician rated							
MINI-KID	X						
CY-BOCS	X		X	X	X	X	X
CGI-S	X		X	X	X	X	X
CGI-I			X	X	X	X	X
CGAS	X		X	X	X	X	
iiPAS^a^		Week 8	X				
PEAS^b^		Weekly	X				
Child rated							
OCI-CV	X	Weekly	X	X	X	X	
MFQ	X		X	X	X	X	
WSAS-Y	X		X	X	X	X	
CHU9D	X		X	X	X	X	
ISI	X		X	X	X	X	
PEAS		Weekly	X				
WAI		Week 2					
TCES		Week 2					
CSQ			X		X		
Parent rated							
ChOCI-R-P	X	Weekly	X	X	X	X	
FAS-SR	X		X	X	X	X	
MFQ	X		X	X	X	X	
WSAS-P	X		X	X	X	X	
AQ-10	X						
TiC-P	X		X	X	X	X	X
Parent strategies		Weekly	X				
WAI		Week 2					
TCES		Week 2					
CSQ			X		X		

Abbreviations: AQ-10, Autism Spectrum Quotient; CGAS, Children’s Global Assessment Scale; CGI-I, Clinical Global Impression–Improvement; CGI-S, Clinical Global Impression–Severity; ChOCI-R-P, Children's Obsessional Compulsive Inventory Revised-Parent version; CHU9D, Child Health Utility 9D; CSQ, Client Satisfaction Questionnaire; CY-BOCS, Children's Yale-Brown Obsessive Compulsive Scale; FAS-SR, Family Accommodation Scale–Self Rated; iiPAS, Internet Intervention Patient Adherence Scale; ISI, Insomnia Severity Index; MFQ, Mood and Feelings Questionnaire; MINI-KID, Mini International Neuropsychiatric Interview for Children and Adolescents; OCI-CV, Obsessive-Compulsive Inventory–Child Version; PEAS, Patient EX/RP Adherence Scale; TCES, Treatment Credibility and Expectancy Scale; TiC-P, Trimbos/iMTA Questionnaire for Costs Associated With Psychiatric Illness; WAI, Working Alliance Inventory; WSAS-P, Work and Social Adjustment Scale–Parent version; WSAS-Y, Work and Social Adjustment Scale–Youth version.

^a^ Score for iiPAS is measured in the stepped care internet-delivered cognitive behavior therapy group.

^b^ Clinician-rated PEAS is assessed during face-to-face cognitive behavior therapy in the gold standard group.

Secondary clinician-rated outcome measures are the CGI Severity and Improvement scales (CGI-S and CGI-I)^23^ and CGAS.^24^ Severity of OCD symptoms is measured using the child-rated Obsessive-Compulsive Inventory–Child Version (OCI-CV)^31^ and the Children's Obsessional Compulsive Inventory Revised–Parent version (ChOCI-R-P).^32^ Family accommodation behaviors are measured using the Family Accommodation Scale–Self Rated (FAS-SR).^33^ Impairment of functioning due to OCD is assessed using the Work and Social Adjustment Scale–Youth version and Parent version (WSAS-Y and WSAS-P), which are adaptations of the Work and Social Adjustment Scale.^34^ Depressive symptoms are assessed using the short version of the Mood and Feelings Questionnaire (MFQ).^35^ The Insomnia Severity Index (ISI)^36^ is used to quantify the severity of insomnia. The Child Health Utility 9D (CHU9D)^37^ is used as a measure of quality of life and for the estimation of quality-adjusted life years. The Trimbos/iMTA Questionnaire for Costs Associated With Psychiatric Illness (TiC-P),^38^ which measures resource use of health care, social support and assistance, medication use, informal care by parents, and school and work absenteeism over the last 3-month period, is administered to the parents. The Autism Spectrum Quotient (AQ-10)^39^ is used as a baseline measure of autistic symptoms rated by the parent. Other measures are the Treatment Credibility and Expectancy Scale (TCES)^40^ to measure how credible participants perceive the treatment to be, the Working Alliance Inventory (WAI)^41^ to measure working alliance, and the Client Satisfaction Questionnaire (CSQ-8)^42^ to assess participants’ satisfaction with treatment. To measure treatment adherence, the Patient EX/RP Adherence Scale (PEAS)^43^ is used to investigate the amount of exposure and response prevention exercises performed weekly (administered as both a clinician-rated and self-reported measure during face-to-face CBT and as a self-rated version during ICBT), and the clinician-rated Internet Intervention Patient Adherence Scale (iiPAS)^44^ is used only in the ICBT group. To investigate the role of parental behaviors in relation to treatment outcome, questions about parental strategies are administered every week during treatment.

### Safety Procedures

Both children and parents report occurrence of adverse events or undesirable treatment effects in the middle of the course of treatment, after the course of treatment, and at the 6-month follow-up. Adverse events are assessed in the online platform where both the child and the parent report and describe the event and its impact on the child. Reported incidents are categorized depending on severity and frequency in line with GCP. All adverse event assessments will be cross-checked by KTA.

All participants are monitored by the study personnel throughout the study period to ensure participant safety. Therapists and assessors have routines for situations of potential risk. Serious adverse events (ie, events that result in death, suicide attempt, serious violent incident, or admission to the hospital) are documented and monitored by KTA. In case a participant’s condition deteriorates during treatment, the therapist does an immediate in-depth assessment of the symptoms and discusses this with the principal investigator to determine necessary actions. For patient safety reasons, an item to assess suicide risk is added to the child-rated MFQ, which is administered before and after treatment and at 3-month, 6-month, and 1-year follow-ups. If the participant indicates suicidal ideation or has a total score greater than 13 on the child- or parent-rated MFQ (total score ranging from 0-26), the therapist will initiate an assessment of suicide risk.

### Statistical Analysis

#### Baseline Data

Statistical analyses will be conducted under the guidance of the Karolinska Institutet Biostatistics Core Facility. Descriptive statistics with sample characteristics will be presented for each group at baseline. There will not be any formal testing for between-group differences on any baseline variable.^45^ However, we will test for possible site differences at baseline.

#### Outcome Data

The only previous pediatric OCD trial using a noninferiority design used −5 points on the primary outcome measure (CY-BOCS) as a noninferiority margin with an SD of 8.^28^ In the present study, we set the noninferiority margin to −4 points to gain greater precision. The noninferiority analysis will be based on the CY-BOCS score at the primary end point (6-month follow-up) and determined by the confidence interval around the mean difference between the 2 groups. The estimated enrollment is 152 participants, which gives an estimated power greater than 95%, allowing for possible data attrition (10% based on experience from our previous trials). The power is estimated based on each participant being observed at 4 points (baseline, after treatment, 3-month follow-up, and 6-month follow-up). We used a linear random intercept model with 1000 bootstrap samples using the following values, which were based on individual-level data from a previous study of therapist-guided ICBT for adolescents with OCD that used 3 repeated observations^14^: intercept (22.45), slope (−0.37 per week), SD of the random effect (4.03), and SD of the residual error (3.94). In other words, if the stepped care ICBT approach is truly noninferior to the gold-standard treatment, 152 patients will give an estimated probability greater than 95% that the upper limit of a 1-sided 95% confidence interval (or, equivalently, a 90% 2-sided confidence interval) will be below the noninferiority limit of 4 points on the CY-BOCS. No specific noninferiority margin is defined for the secondary outcome measures, but we expect no statistically significant difference between the groups on these measures.

Data will be analyzed according to the participants’ original treatment allocation in line with intention-to-treat principles. Data analysis on the continuous outcome measures will be performed with mixed-effect regression analyses for repeated measures with maximum likelihood estimation. The model will include fixed effects of time, treatment group, and site and an interaction effect of treatment group by time, as well as random intercept and random slope to account for individual differences. In line with our secondary aim, a model with an interaction effect of treatment group, time, and source of referral will be implemented to explore whether source of referral is a moderator of treatment effect. Ordinal data will be analyzed with ordinal regression, binary data will be analyzed with logistic regression, and paired binary data will be analyzed with McNemar tests. Logistic regression will be used to test whether the blinded assessor’s guesses on treatment allocation are better than chance. Within- and between-group effect sizes will be reported as Cohen *d*. If the proportion of missing data is greater than 10%, we will perform multiple imputation with 10 extra data sets.^46^ In a first sensitivity analysis, we will compare the results of the complete-case analysis with those of the multiple-imputed data sets. If the results are inconsistent, we will investigate the reasons of the nonresponse and discuss their possible impact on the study findings. In a second sensitivity analysis, we will exclude the participants who violate protocol (eg, start medication in the middle of the trial) and investigate any inconsistencies with the results from the main analysis. Additional analysis of the 1-year, 2-year, and 5-year follow-up data will evaluate whether the treatment gains are maintained long term.

#### Cost-effectiveness Analysis

In our cost-effectiveness analysis, we will compare costs and health outcomes between stepped care ICBT and gold-standard CBT. Costs will be collected from 2 different perspectives: a health care organization perspective and a societal perspective. Costs within the health care organization perspective include recorded therapist time in the ICBT treatment and on face-to-face CBT sessions (including time for preparation and traveling), telephone calls, and administration. In the analysis from a societal perspective, other health care costs (other health care utilization and medication use) as well as indirect costs (eg, informal care, productivity loss associated with school and work absenteeism) captured by the TiC-P will be included.^38^ Our hypothesis is that there will be a difference between the 2 groups in treatment costs from the health care organization perspective, but we expect no difference in other costs.

Two types of analyses will be conducted on each of the 2 perspectives: a cost-effectiveness analysis and a cost-utility analysis. In the cost-effectiveness analysis, both number of responders and remitters at primary end point (6-month follow-up) will be used as the health outcome. We will use expert consensus criteria to define treatment response and remission.^30^ A responder is defined as at least 35% reduction on the CY-BOCS and a CGI-I score of 1 (very much improved) or 2 (much improved). Remission is defined as a score of less than or equal to 12 on the CY-BOCS and a CGI-S rating of 1 (normal, not at all ill) or 2 (borderline mentally ill). In the cost-utility analysis, the outcome will be quality-adjusted life years, according to international standards for cost-effectiveness analyses.^47,48^ Quality-adjusted life-years will be calculated using the area under the curve method.^49^ Costs will be estimated by appropriate regression analyses testing alternative link functions and distributions.^50^ Nonparametric bootstrapping with 5000 iterations will be carried out, pairing up differences in costs with differences in outcomes. As a global cost-effectiveness estimate, we will present the incremental cost-effectiveness ratio (difference in costs between the 2 groups divided by the difference in effect),^51^ as well as a visual presentation of cost-effectiveness planes. The time frames for the cost-effectiveness analyses will be from baseline to the 6-month follow-up, and from baseline to the 1-year follow-up.

### Ethics and Dissemination

The study is conducted according to the Declaration of Helsinki^52^ and GCP standards. All investigators and therapists involved in the study will attend a course in GCP that KTA arranges. The study results will be reported following the Consolidated Standards of Reporting Trials Extension (CONSORT Extension) reporting guideline 2010 statement for noninferiority trials,^53^ and the Consolidated Health Economic Evaluation Reporting Standards (CHEERS) reporting guideline.^54^ The results will be published in peer-reviewed academic journals, presented at scientific conferences, and communicated to the participants and patient organizations.

### Trial Status

Recruitment started October 6, 2017, and was completed in May 24, 2019. Results from the primary end point will be available by May 2020. The naturalistic follow-up will continue to 2025. No interim analyses are planned, and there are no prespecified stopping rules.

## Conclusions

If, as expected, the stepped care ICBT approach is noninferior to the gold standard of CBT but uses fewer resources, it will provide an opportunity to increase the availability of evidence-based treatment for children and adolescents with OCD. The next challenge would be to implement the proposed stepped care model in routine clinical services.

## Notes

Supplement. **Trial Protocol**
