# Supplementary material for: Stepped Care Internet-Delivered vs Face-to-Face Cognitive-Behavior Therapy for Pediatric Obsessive-Compulsive Disorder: A Trial Protocol for a Randomized Noninferiority Trial
Source: JAMA Netw Open. 2019 Oct 23;2(10):e1913810. doi: 10.1001/jamanetworkopen.2019.13810 (PMC6820063; doi:10.1001/jamanetworkopen.2019.13810)
Supplement: Supplement. — Trial Protocol [file jamanetwopen-2-e1913810-s001.pdf]

TRIAL PROTOCOL

Title of the trial	Stepped care internet-delivered versus face-to-face cognitive-behavior therapy for pediatric obsessive-compulsive disorder: a randomized controlled non-inferiority trial
Version and date of protocol	Version 4, 2019-04-29
Sponsor	Stockholm County Council
Ethical review number	2017/1070/31-1
Trial registration number	NCT03263546
Sites	Two-site: two specialist pediatric OCD clinics located at the Child and Adolescent Psychiatry Research Centre in Stockholm, and Sahlgrenska University Hospital in Gothenburg, Sweden
Principal investigator:	Associate professor Eva Serlachius: eva.serlachius@ki.se<br><br>Child and Adolescent Psychiatry Research Centre, Gävlegatan 22, SE-113 30 Stockholm Sweden.

Principan Investigator: Associate professor Eva Serlachius

Signature:

Date: 2019-04-29

VERSION HISTORY

Version number	Version date	Reason for Change
1	2017-05-24	Version submitted to the Regional Ethical Review Board in Stockholm
2	2017-09-05	Changed self-rated measure of depression from CDI-S to MFQ.<br><br>Added ISI as self-reported measure of insomnia severity.<br><br>Self- and parent-reported measures are administered prior to baseline clinician assessment, instead of after the clinician visit.<br><br>All changes are approved by the Ethical Review Board (2017/152-32) and documented in the Karolinska Institutet Electronic Lab Notebook with date and time stamp.
3	2018-05-14	Removal of weekly self-reported measures for the non-responders who receive additional CBT between 3-month and 6-month follow-up to avoid participant burden.<br><br>Documented in the Karolinska Institutet Electronic Lab Notebook with date and time stamp.
4	2019-04-29	Updated power-calculation and statistical analyses plan after consultation with Professor Matteo Bottai.<br><br>Updated plan for cost-effectiveness analyses after consultation with Associate professor Inna Feldman.<br><br>TCES is administered at week 2, previously reported at week 3 by mistake.<br><br>All changes documented in the Karolinska Institutet Electronic Lab Notebook with date and time stamp.

TABLE OF CONTENT

SUMMARY 3

BACKGROUND 4

OBJECTIVE 6

RESEARCH QUESTIONS 6

PROJECT DESCRIPTION 7

DESIGN 7

STUDY SITES 9

POWER ANALYSIS 9

INCLUSION AND EXCLUSION CRITERIA 9

MEASURES 10

Clinician rated measures 10

Child-rated measures 11

Parent-rated measures 12

Other measures 12

PROCEDURE 15

BASELINE ASSESSMENT (W0) 15

INTERVENTIONS (W1-W16) 15

Internet-delivered CBT 15

Face-to-face CBT 16

Adherence 16

Measures 16

POST-TREATMENT ASSESSMENTS (W16-W18) 16

3-MONTH FOLLOW-UP 17

ADDITIONAL FACE-TO-FACE CBT (FOR NON-RESPONDERS) 17

6-MONTH FOLLOW-UP (PRIMARY ENDPOINT) 17

1-YEAR FOLLOW-UP 17

2- AND 5-YEAR FOLLOW-UP 17

QUALITY CONTROL 17

LINKAGE WITH REGISTERS 18

STATISTICAL ANALYSIS 19

TIME PLAN 20

ETHICAL CONSIDERATIONS 21

IMPLICATIONS 21

REFERENCES 23

SUMMARY

Obsessive-compulsive disorder (OCD) is a major psychiatric disorder affecting approximately 2% of the population. Most patients have an onset of symptoms in childhood, around 12 years of age. OCD is associated with severe impairments in academic, social and family functioning. Quality of life tends to be poor.

Cognitive behaviour therapy (CBT), the first line treatment for the disorder, is effective for about 70% of paediatric OCD patients. The main obstacle is that therapist accessibility is low. CBT for OCD is a highly specialised and resource intensive treatment and innovative solutions are needed to satisfy the large demand for this treatment. Internet-based CBT (ICBT) is a new way of increasing CBT accessibility and treatment capacity. Our research group has evaluated ICBT for adolescents with OCD in one pilot study and a randomized controlled trial (RCT), and for children in one pilot study, all with encouraging results.

Building on our previous work, the current randomized non-inferiority trial will evaluate if ICBT can be delivered in a stepped care fashion with maintained efficacy but reduced costs compared to traditional face-to-face CBT, and thus resulting in increased cost-efficacy. One hundred and fifty two patients will be randomized to either stepped care ICBT or to gold standard face-to-face CBT. One group (stepped care) will be first offered ICBT for 16 weeks. Non-responders at the 3-month follow-up will receive additional face-to-face CBT. The other group (gold standard treatment) will be randomized directly to receive face-to-face CBT for 16 weeks. Non-responders will, as in the other group, receive additional face-to-face treatment after the 3-month follow-up (as usually implemented in regular psychiatric care). The primary endpoint will be at 6-month follow-up. Additional follow-ups will be at one-, two- and five years. A full health economic evaluation will be conducted.

If, as predicted, the proposed stepped care model is shown to be non-inferior to, and more cost-effective than, gold standard treatment, we will aim to implement it in our specialist clinic within the next five years.

BACKGROUND

OCD is a major psychiatric disorder affecting approximately 1% of young people and 2% of adults.[1] OCD is a leading cause of disability in the developed world and, in young people, is associated with severe impairments in academic, social and family functioning[2] and increased risk of future mental health problems in adulthood.[3] CBT is effective for about 70% of paediatric OCD patients with an on average large effects on symptom reduction (g = 1.21), treatment response (relative risk [RR] = 3.93), and symptom/diagnostic remission (RR = 5.40).[4] Despite expert guidelines recommending CBT, OCD is often not adequately diagnosed and treated, and a majority of OCD sufferers do not get access to evidence-based treatments.[5] Some possible barriers include low numbers of properly trained CBT therapists within the health care system,[6 7] financial and logistic (e.g. geographical) barriers to treatment seeking as well as embarrassment due to symptoms.[8-10]

Internet-delivered interventions have a high potential to overcome barriers to treatment and make CBT more accessible. ICBT for adult patient populations has gained increasing empirical support during the last decade and shown efficacy for a number of different psychiatric conditions.[11] In terms of content, ICBT mimics traditional CBT, the only difference being the way care is delivered. In ICBT, the patient, instead of going to a clinic, logs on to a secure website and works with written self-help materials and homework assignments, which are closely monitored by a clinician. As in regular CBT for OCD, the main treatment component is exposure with response prevention (ERP), which teaches the patient to challenge the symptoms despite increased discomfort, and over time leads to decreased symptoms and impairment.[12] The main advantages with ICBT are that it is highly accessible and that the required therapist times can be reduced to a fraction of what is necessary for face-to-face CBT.[11]

ICBT for adults with OCD has been evaluated extensively in six randomized controlled trials compared with both wait-list and attention-control conditions[13-18], with maintained long-term effects up to two years after treatment.[19 20] Currently, there is an on-going non-inferiority trial for adults with OCD comparing ICBT with and without therapist support to face-to-face CBT (ClinicalTrials.gov ID NCT02541968).

Previously, our research group has conducted a cost-effectiveness analysis of ICBT for adult OCD.[21] Results are summarised in **Figure 1**. The X-axis in this graph is the “willingness-to-pay” i.e. the additional cost that society is willing to pay for one additional remission of OCD by offering ICBT instead of support therapy. The Y-axis is the probability of ICBT being cost-effective given the different willingness-to-pay scenarios. When using a societal perspective (including all type of costs such as sick-leave, unemployment, doctors visits etc.),

ICBT had a 90% chance of being cost-effective given a willingness-to-pay of $2600 for a remitted case of OCD. When narrowing the perspective to health care unit costs (i.e. treatment cost only), the corresponding figure was $900. Thus, ICBT is likely to be a cost-effective treatment that can provide society with additional remissions of OCD.

FIGURE 1. HEALTH ECONOMIC DATA FROM A PREVIOUS ADULT OCD ICBT TRIAL.[21]

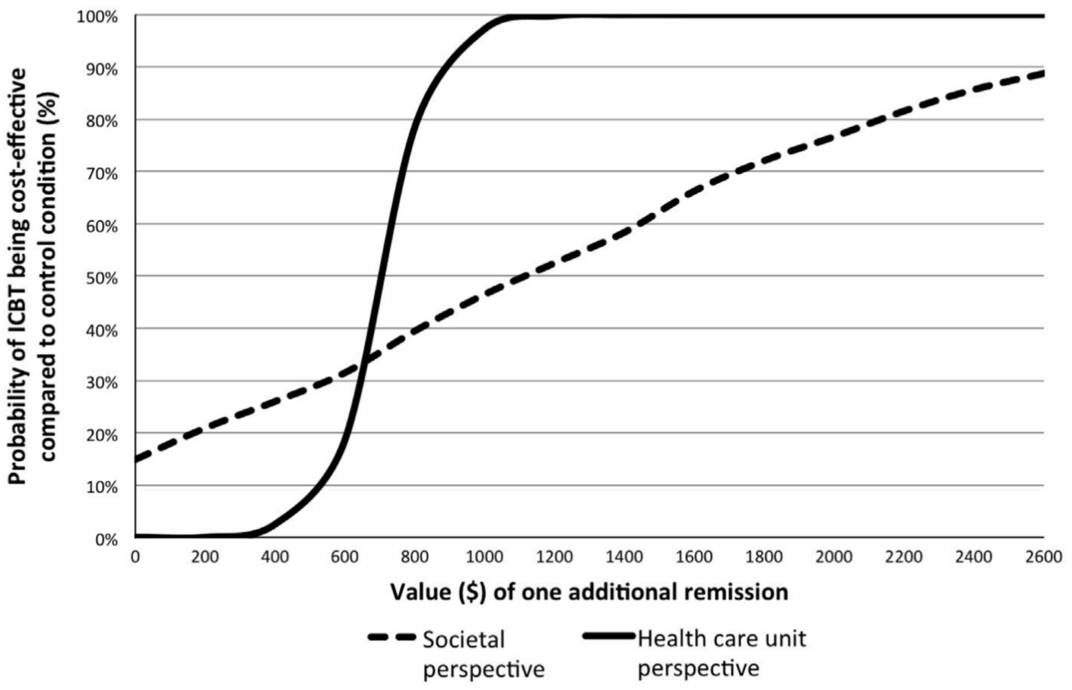

Since 2010, our research group has made substantial efforts to develop and test ICBT for children and adolescents with various conditions, with published results on ICBT for children with anxiety disorders,[22 23] adolescents with OCD,[24 25] and adolescents with functional gastrointestinal disorders (FGID).[26 27] We have also completed, but yet unpublished studies for children with OCD (ClinicalTrial.gov NCT02663167), children and adolescents with Tourette's Disorder and Chronic Tic Disorder (ClinicalTrial.gov NCT02864589), and adolescents with social anxiety disorder (ClinicalTrial.gov NCT02576171), manuscripts in preparation. Currently we have on-going studies for adolescents with excessive worry (ClinicalTrial.gov NCT02978963), children with functional abdominal pain disorders (ClinicalTrial.gov NCT02873078), and adolescents with non-suicidal self-harm (ClinicalTrial.gov NCT02697019).

For OCD, we first developed an ICBT protocol for adolescents. Results from a first pilot study (N = 21) showed that ICBT was indeed an acceptable and effective intervention for adolescents with OCD, with 57% treatment responders at post-treatment.[25] In a subsequent RCT,[24] we randomized 67 adolescent OCD patients to either ICBT or to a waiting-list.

Results showed that ICBT was superior to the waiting list with a between-group effect size at post-treatment of $d = 0.64$. Using strict consensus criteria, 27% percent of the participants in the ICBT group were classified as responders, compared to 0% in the waiting-list. At the 3-month follow-up, the amount of responders in the ICBT group increased to 32%. The time to treat each patient was only a third of traditional face-to-face CBT which is in line with our health-economical evaluations of ICBT for adults with OCD.[21] We have also developed and evaluated the feasibility and efficacy of ICBT for younger children with OCD (ages 7-11) in a pilot study (manuscript in preparation).[28] The study showed that ICBT also is a feasible and effective treatment for the younger age group, with 64% treatment responders at 3-month follow-up.

Thus, our previous work has shown that ICBT is an effective treatment for the paediatric OCD population with the potential to significantly reduce the time needed to treat each patient. However, the effect sizes obtained in the RCT were somewhat lower than would be expected in face-to-face CBT. Based on our data, it seems plausible that many patients will probably benefit from ICBT if given as first line treatment but that approximately 50% will require additional treatments (that is, face to face CBT). Given the apparent advantages with ICBT from an accessibility and resource use perspective, the next logical step is to evaluate if a stepped care approach, where ICBT is offered in the first step and face-to-face CBT is only offered to those who fail to respond sufficiently to ICBT.

OBJECTIVE

CBT is the first line treatment for young people with OCD but this highly specialized treatment is not available to most sufferers. There is an urgent need to make this treatment more accessible to patients. The primary objective of this study is to test the effectiveness of therapist-guided ICBT in a stepped care model compared to gold standard treatment and evaluate the cost-effectiveness.

RESEARCH QUESTIONS

Our primary research question is if therapist-guided ICBT in a stepped-care approach is an effective and cost-effective treatment in reducing OCD symptoms compared to gold standard treatment (face-to-face CBT).

Our hypotheses are

a. An ICBT stepped care approach will be non-inferior to gold standard treatment (face-to-face CBT) in reducing OCD symptoms.

b. The stepped care approach will be associated with lower costs for the health care provider (such as reduced therapist and administration times) compared to gold standard treatment, but we expect no differences in other costs (other service utilization, medication use, informal care, productivity loss associated with work and school absence).
c. Self-referred patients will benefit significantly better than referred patients regarding outcome in the ICBT group, but there will not be any difference in the face-to-face CBT group.

We also aim to study whether ICBT is more suitable for certain subgroups of patients, look at predictors and moderators of outcome, conduct mediation analysis, as well as investigate the long-term efficacy of the treatment.

d. Study whether ICBT is more suitable for certain subgroups of patients in terms of baseline characteristics with machine-learning. Explore which patient characteristics predict positive outcome in ICBT for children and adolescents with OCD.
e. Do mediation analysis to see if the use of the treatment strategies mediates the treatment effect, and potentially does so differently in the two intervention conditions. More specifically, if the use of the treatment strategies exposure and response prevention measured with PEAS and the use of parent strategies mediate treatment outcome?
f. Investigate the long-term effectiveness of ICBT in reducing OCD-symptom severity.

PROJECT DESCRIPTION

DESIGN

Two-site randomized non-inferiority trial. Experimental condition is “stepped care” vs. “gold standard” treatment for children and adolescents with OCD. Half of the participants (“stepped care”) will be randomized to first receive ICBT for 16 weeks. Non-responders at the 3-month follow-up (due to expected additional treatment effects between post-treatment and follow-up) will receive additional face-to-face CBT. The other half of the sample (“gold standard”) will be randomized directly to face-to-face CBT for 16 weeks. Non-responders will, as in the other arm, receive additional treatment after the 3-month assessment point. All participants will be followed up 6- month after the first treatment period. The primary endpoint is set to the 6-month follow-up (see **Figure 2**). Additional follow-ups will be conducted one-, two- and five-years after finished treatment. Participants will also be asked to consent for linkage with registers, including retrieving information about both the patient and the parents.

FIGURE 2. STUDY DESIGN

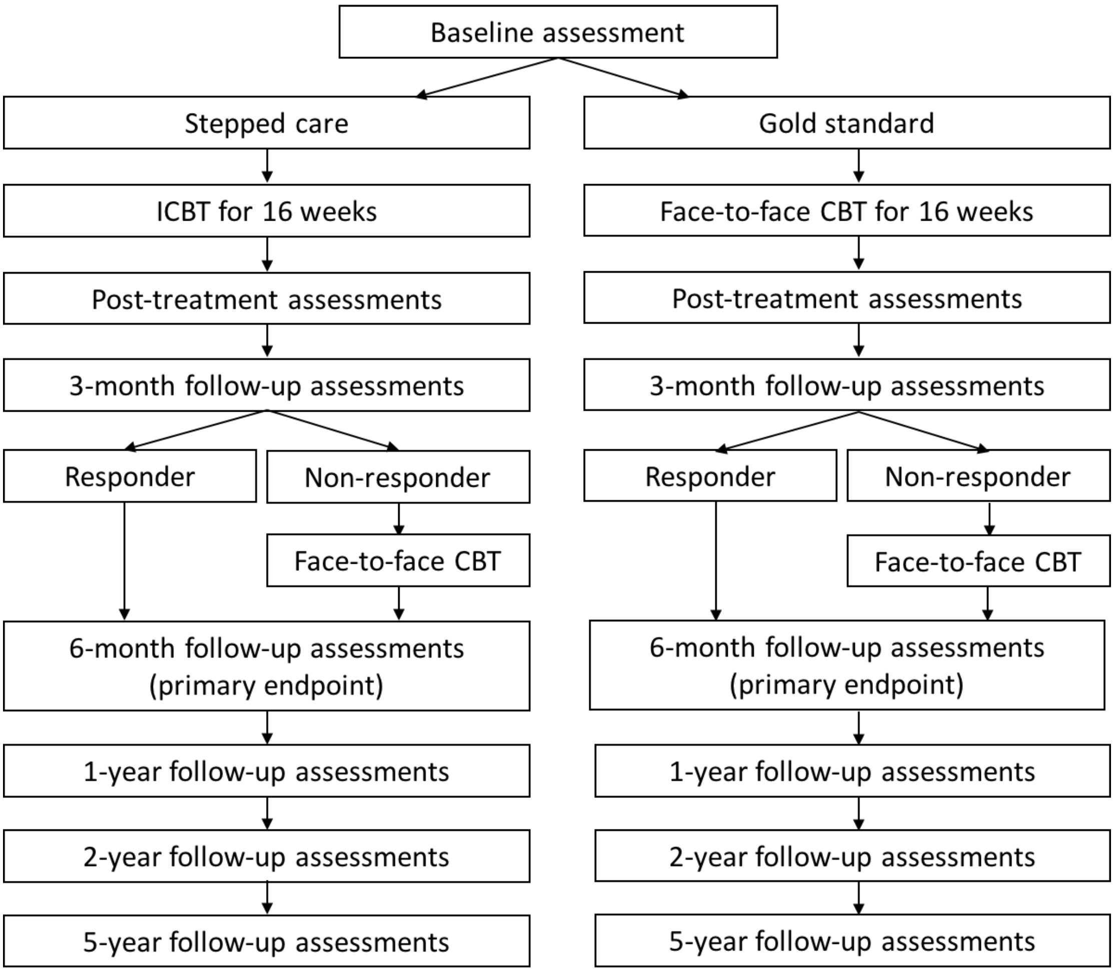

STUDY SITES

The project is a collaboration between the Department of Clinical Neuroscience at Karolinska Institutet, the Child and Adolescent Mental Health Service (Barn- och ungdomspsykiatrin, BUP) in Stockholm and the Child and Adolescent Mental Health Service (BUP) in Gothenburg.

Study participants are assessed and treatment at either a specialized OCD unit (BUP OCD) or a clinical research unit (BUP klinisk forskningsenhet) within the Child and Adolescent Mental Health Service in Stockholm, or a specialized OCD unit (BUP Specialmottagning) within the Child and Adolescent Mental Health Service in Gothenburg. Both BUP OCD and BUP klinisk forskningsenhet are located within the premises of CAP Research Center, Gävlegatan 22, 113 30 Stockholm, Sweden. BUP Specialmottagning is located at Lindhultsgatan 23, 416 74 Gothenburg, Sweden.

POWER ANALYSIS

The only previous pediatric OCD trial employing a non-inferiority design used -5 points on the primary outcome measure (CY-BOCS) as non-inferiority margin with a standard deviation of 8.[29] In the present study, we set the non-inferiority margin to -4 points. The non-inferiority analysis will be based on the CY-BOCS score at the primary endpoint (6-month follow-up). The estimated enrollment of participants is set to 152 participants, which gives an estimated power well above 95%, allowing for possible data attrition (10% based on experience from our previous trials). The power is estimated based on each participant being observed at four time points (baseline, post-treatment, 3-month and 6-month follow-up). We used a linear random intercept model with 1000 bootstrap samples using the following values, based on individual-level data from a previous study of therapist-guided ICBT for adolescents with OCD that used three repeated observations:[24] intercept (22.45), slope (-0.37), standard deviation of the random effect (4.03), and standard deviation of the residual error (3.94). In other words, if the stepped care ICBT approach is truly non-inferior to gold standard treatment, 152 patients will give an estimated probability above 95% that the upper limit of a one-sided 95% confidence interval (or equivalently a 90% two-sided confidence interval) will be below the non-inferiority limit of 4 points on the CY-BOCS. No specific non-inferiority margin is defined for the secondary outcome measures.

INCLUSION AND EXCLUSION CRITERIA

Inclusion criteria are:

a) Primary DSM-5 diagnosis of OCD[30]
b) Total score of ≥16 on the Children's Yale-Brown Obsessive-Compulsive Scale (CY-BOCS)[31]

c) Age between 7 and 17 years
d) Ability to read and write Swedish and with access to a computer and use of internet.
e) Participants on psychotropic medication must had been on a stable dose for the last 6 weeks prior to baseline assessment. If the child is on medication for OCD, they will be requested to stay on stable doses for the duration of the trial.

Exclusion criteria are:

a) Organic brain disorders, global learning disabilities, autism spectrum disorder, psychosis, bipolar disorder and severe eating disorder. However, most other comorbidities (e.g. depression, anxiety) will be allowed, as these are frequent and should not interfere with the treatment.
b) Suicidal ideation
c) Not able to understand the basics of the ICBT self-help material, patient being housebound or in need of intensive- or in-patient treatment.
d) Having completed a course of CBT for OCD within the last 12 months (defined as at least 5 sessions CBT including exposure and response prevention).
e) Children with ongoing psychological treatment for OCD or another anxiety disorder will not be included in the study.

MEASURES

An overview of the measures that will be used in the study is presented in table 1.

Clinician rated measures

Children's Yale-Brown Obsessive-Compulsive Scale (CY-BOCS)[31] is a semi-structured clinician administered interview for assessment of symptom severity in paediatric OCD. CY-BOCS is the primary outcome measure in this study.

We will take extensive steps to avoid any measurement bias on the CY-BOCS:

1) All clinicians will practice together how to administer the CY-BOCS on at least four different case examples.
2) We will then test inter-rater reliability one three additional case examples and repeat this procedure until the inter-rater reliability intraclass correlation coefficient is at least 0.85.
3) Assessors will be independent and blinded to treatment allocation throughout the duration of the trial to the 6-month follow-up.
4) To ensure the integrity of the blinding procedure, participants will be instructed not to mention which treatment they have received during the assessment interviews.
5) If the participant mentions the treatment allocation and the assessment is not blind, the part where the blinding is broken will be cut out from the audiofile and another

person will re-rate the CY-BOCS and the we will change rater for the following assessments.

6) After completing the interviews, the blind rater will also guess allocation status for each participant (we expect that this guess will not be better than chance).

Clinical global impression severity and improvement (CGI-S/I).[32] CGI-S is a brief clinician rating of symptom severity and ratings are on a seven graded scale from 1 = "no symptoms" to 7 = "extreme symptoms". CGI-I is a rating of the patient's symptom severity change relative to the baseline assessment and is rated from 1 = "very much improved" to 7 = "very much worse". CGI-S will be rated at all assessments point and CGI-I will be rated at post-treatment and all follow-up assessments. All clinicians will practice rating CGI-S/I together and inter-rater reliability will be tested.

Children's global assessment scale (C-GAS)[33] is used by clinicians to assess global functioning on a scale from 0 to 100 in children and adolescents, with higher scores indicating higher functioning.

Mini International Neuropsychiatric Interview for Children and Adolescents (MINI-KID).[34] MINI-KID is a short structured diagnostic interview for DSM-IV and ICD-10 psychiatric disorders in children and adolescents. It will be used to assess OCD diagnosis and psychiatric comorbidity at baseline. All clinicians will receive training in MINI-KID prior to the study.

Patient EX/RP Adherence Scale (PEAS)[35] is designed to measure patients adherence to the treatment strategies exposure and response prevention. It will be clinician-administered weekly in the face-to-face CBT treatment.

Internet Intervention Patient Adherence Scale (iiPAS) is an adaptation of PEAS,[35] and contains items that are more oriented towards internet-delivered treatments. It is developed by our research group, and will be a rated at mid- and post-treatment for those patients that receive ICBT.

Child-rated measures

Obsessive-compulsive inventory – child version (OCI-CV).[36] Self-reported measure of OCD-symptoms. It will be administered at each assessment as well as weekly during treatment for both treatment groups.

The Child Health Utility 9D (CHU 9D)[37] is a self-rated measure of health-related quality of life. The scale contains nine dimensions with five levels that represents degrees of severity within each dimension.

Patient EX/RP Adherence Scale (PEAS)[35] an adaptation of the clinician-rated PEAS will be administered weekly in both treatment conditions. The ratings will correlated with the clinician-rated PEAS in the face-to-face group to validate the self-rated version.

Insomnia Severity Index (ISI)[38] is a 7-item scale measuring sleep problems. Each item is rated on a 4-point scale. ISI will be administered at each assessment point.

Parent-rated measures

Children's Obsessional Compulsive Inventory Revised – parent version (ChOCI-R-P)[39] is a parent-rated measure of the child's OCD-syptom severity. It will be administered at each assessment as well as weekly during treatment for both treatment groups.

Family accommodation scale for obsessive-compulsive disorder – self-rated version (FAS-SR)[40] is a 19-item scale that is designed to assess family accommodation due to the child's OCD, and will be administered at each assessment point.

Autism Spectrum Quotient (AQ-10)[41] is a brief 10-item scale that is used to screen for autistic symptoms. There are separate versions for children 4 – 11 years and adolescents 12 – 17 years. It will be administered at baseline assessment and used as a predictor for outcome.

Trimbos/iMTA questionnaire for Costs associated with Psychiatric Illness – Child version (TiC-P).[42] TiC-P is used to measure consumption of health care, costs associated with mental illness and production loss among parents due to psychiatric problems in the child. It will be used for the health economy analysis. TiC-P will be administered to parents at all assessment points.

Other measures

Mood and Feeling Questionnaire – child and parent version (MFQ)[43] is a 13-item parent-rates measure if the child's depressive symptoms, and will be administered at each assessment point. An additional item will be added to the child version to assess suicide risk.

Work and social adjustment scale – youth version (WSAS-Y)[44 45] is a 5-item child- and parent-rating scale measuring impairment and functioning in psychiatric patients.

Treatment credibility and expectancy – child and parent version (TCES)[46] is a 5-item scale measuring the degree to which participants rate the treatment credibility and their expectancies regarding the treatment outcome. The questions will be administered to both children and parents at week 2 in the first course of treatment for all participants.

Working alliance inventory – child and parent version (WAI)[47] is a 6-item scale measuring the participants perceived working alliance with their therapist, and is used to control for non-specific therapeutic effects between the different treatment conditions. The questions will be

administered to both children and parents at week 2 in the first course of treatment for all participants.

Client Satisfaction Questionnaire – child and parent version (CSQ-C/P)[48] is a 8-item scale that is used to measure satisfaction with the treatment. It will be administered to both children and parents at post-treatment and at 6-month follow-up.

Unintended treatment effects. During the intervention phase, the study participants (children and parents) will be asked to fill in a web-based form on the occurrence of any unintended treatment effects or adverse events as a reaction to or consequence of the intervention. Frequency of those incidents will be reported in the publication of the study. Questions will be asked at mid-treatment, post-treatment and at 6-month follow-up.

Questions about parent strategies will be administered weekly to the parents to investigate if the use of the presented strategies mediates the outcome.

Demographic and background variables. During the self-report procedure and at the clinician-visit at baseline, questions about parental educational level and occupational status, country of birth for the parents and child, prior treatment history (e.g. previous CBT or medication including response), and onset of OCD-symptoms will be recorded. Information on how the participants got in contact with the study will be registered (e.g. via media, referral, or recommendation).

TABLE 1. LIST OF MEASURES AND ASSESSMENT POINTS.

Measure \ Timeline	Baseline assessment	During treatment	Post-treatment assessment	3-month follow-up	Face-to-face CBT for non-responders	6-month follow-up	1-year follow-up	2- and 5-year follow-up (via telephone)
**Clinician-rated**								
Demographic data	X							
MINI-KID	X							
CY-BOCS	X		X	X		X	X	X
CGI-S	X		X	X		X	X	X
CGI-I			X	X		X	X	X
CGAS	X		X	X		X	X	
iiPAS (ICBT group)		Week 8	X					
PEAS (F2F CBT)		Weekly	X		X			
**Child-reported**								
OCI-CV	X	Weekly	X	X		X	X	
MFQ-C	X		X	X		X	X	
EWSAS-C	X		X	X		X	X	
CHU9D	X		X	X		X	X	
ISI	X		X	X		X	X	
PEAS		Weekly	X					
WAI		Week 2						
TCES		Week 2						
CSQ-C			X			X		
Unintended treatment effects		Week 8	X			X		
**Parent-reported**								
Demographic data	X							
ChOCI-R-P	X	X	X	X		X	X	
FAS-SR	X		X	X		X	X	
MFQ-P	X		X	X		X	X	
EWSAS-P	X		X	X		X	X	
AQ-10	X							
TiC-P	X		X	X		X	X	X
Parent strategies		Weekly	X					
WAI		Week 2						
TCES		Week 2						
CSQ-P			X			X		
Unintended treatment effects		Week 8	X			X		

PROCEDURE

Participants can be self-referred or referred by a clinician (type of referral will be stratified in the randomization procedure). All referred participants will receive written information about the study (including objectives, benefits, risks and requirements imposed by the study).

BASELINE ASSESSMENT (W0)

Participants interested in participating in the study will complete a screening that consists of self and parent administered questionnaires listed above. This is assessed through the online treatment platform. The information will be deleted if the participant is not included in the study.

After completing the screening, a face-to-face interview is conducted with a clinician at one of the clinics. The clinician will a) verify diagnosis, b) assess psychiatric comorbidity, c) decide on inclusion/exclusion and d) assess current OCD severity level. Parallel treatments will be checked through medical records. The psychiatric interview includes the MINI-KID.[34] Informed consent is signed and collected during this visit.

The CY-BOCS interviews are recorded for assessment of inter-rater reliability (informed consent is collected prior to recording). Patients are randomly assigned to either stepped care or gold standard. Randomisation will be stratified by source of referral (self- vs clinic), and by participant age (child 7-12 years vs adolescent 13-17 years). Families who decline participation will be offered regular treatment at our clinic or referred to other clinical services, depending on each individual's needs.

Participants will start treatment within two weeks after inclusion. If the treatment start is delayed more than two weeks, the CY-BOCS will be re-administered over the telephone. If the OCD-symptoms have decreased so the patient does not meet inclusion criteria at this time point (a total score of <16 on the CY-BOCS), the participant will enter follow-up instead.

INTERVENTIONS (W1-W16)

Internet-delivered CBT

The ICBT-treatment is completely web-based and involves both the child/adolescent and the parents. The program spans over 16 weeks and includes psychoeducation, rationale for exposure, homework assignments, and relapse prevention. The content of the program is presented in a varied and engaging manner with reading material, films, animations, illustrations, and exercises. Exercises are aimed at consolidating knowledge, practicing new skills, relating personally to the content or increasing interactivity. During the treatment period, the participants have regular contact with the therapist through written messages in the

online platform or, when necessary, telephone calls. Families have the same therapist during the entire treatment.

Face-to-face CBT

The content in the *face-to-face CBT* is the same as in the ICBT with the difference that it is fully provided by a clinician for 14 1-hour sessions over a period of 16 weeks, according to validated protocols.[17]

Adherence

The therapist adherence to the treatment protocol will be assessed during the intervention. Also, all therapists in the trial will receive continues supervision and discuss the patients on weekly meetings.

In f2f CBT, all sessions will be recorded and a random sample of 20% will be assessed regarding the therapist's competence and adherence to the treatment protocol by using a checklist.

In the ICBT condition, a random sample of 20% of patients and sessions will be assessed using the same checklist as in f2f CBT by reading the correspondence between the patient and the therapist for that specific module.

Measures

OCD-symptoms are assessed weekly by self-rated OCI-CV and parent-rated ChOCI-R-P. In order to perform mediation analysis, the participants will answer weekly questions about the use treatment strategies measured with PEAS, and the parents will answer questions about their use of the presented parent strategies. In order to establish whether the two interventions are perceived by the participants as being equally credible, we will administer the TCES at week 2. WAI will also be administered at week 2 to assess the perceived working alliance. At mid-treatment, questions regarding unintended treatment effects and adverse events will be administered online.

POST-TREATMENT ASSESSMENTS (W16-W18)

The post-treatment assessments will be conducted by independent evaluators masked to treatment condition. The assessment will include all outcome measures listed above. The interview with CY-BOCS will be recorded for assessment of inter-rater reliability. Treatment completion is defined as at least one session (face-to-face CBT) or chapter (ICBT) with exposure and response prevention, as well as using the PEAS to quantify the amount of exposure in both treatment groups.

3-MONTH FOLLOW-UP

Blind rater visit, same procedure as the post-treatment assessments. Patients who are not classified as responders (responder defined as at least 35% reduction on the CY-BOCS and CGI score of 1 or 2)[49] will be offered additional face-to-face CBT (booster CBT) for maximum 12 weeks. Patients who are classified as responders will continue assessment points without any additional treatment.

ADDITIONAL FACE-TO-FACE CBT (FOR NON-RESPONDERS)

The additional face-to-face CBT that are offered to non-responders consist of up to 12 sessions over 12 weeks. The length of each session will be tailored to the individual needs of the patient, and home visits will be made when necessary.

6-MONTH FOLLOW-UP (PRIMARY ENDPOINT)

Blind rater visit, same assessment procedure as the post-treatment assessments. This is the primary endpoint on which the statistical analysis to answer the research questions will be conducted. In this visit, assessment of the patients need of additional care will be conducted, and actions taking accordingly.

1-YEAR FOLLOW-UP

Same assessment procedure as the post-treatment assessments. Patients who still experience significant OCD symptoms will be offered additional multimodal treatment at our specialist clinic or be referred to more appropriate services.

2- AND 5-YEAR FOLLOW-UP

Long-term follow-ups will be done over the telephone two and five years after the treatment finished. The clinician-rated measures CY-BOCS, CGI-S and CGI-I will be administered, and the TiC-P questions will be asked.

QUALITY CONTROL

The trial will be conducted according to good clinical practice. Data quality and any safety aspect of this study will be regularly monitored by Karolinska Trial Alliance (KTA). Randomisation- and masking procedures will be conducted by KTA. Sealed envelopes with randomization number will be stored at the KTA facility. Statistical analyses will be conducted under the guidance of the KI Biostatistics Core facility (www.biostatcore.ki.se).

LINKAGE WITH REGISTERS

Register data from several Swedish registers are linked together using the unique peronal identification number. See Table 2 for registers. The aim with register linkage is to study risk factors for disease, consequences of OCD and long term outcome of treatment. We are aware of the relatively small sample size of this study in order to analyse environmental risk factors for development of OCD. However, in a long-term perspective this material will be part of a bigger effort at our clinic to build a sufficiently large material in order to answer research questions related to these matters.

TABLE 2. REGISTERS

**Register name**	**Register holder**	**Key data**
National Patient Register	EpC	Diagnosis, Hospitalization, Suicide attempts
Causes of Death	EpC	Causes of Death
Prescription register (Läkemedelsregistret)	EpC	All presciptions
Multigeneration register	SCB	Linkage between generations
Marital status changes, county changes and immigration/emigration	SCB	
LISA (Longitudinal integration database for health insurance and labour market studies)	SCB	Income, Highest level of education and employment status
Census, FOB (Folk- och bostadsräkningen)	SCB	http://www.scb.se/Pages/List____257507.aspx<br>Senast 1990
Register of school grades (elementary school and gymnasium)	SCB	Grades
Sick leave, disability	Försäkringskassan	Sick leave, disability pension
VAL data bases: primary care	Karolinska Inst	Diagnoses
The Medical Birth Register	EpC	Birth weight,, APGAR
VAL data bases: psychiatric outpatient care	Karolinska Inst	Diagnoses
Conscript register	Pliktverket	Test results

STATISTICAL ANALYSIS

Data will be analyzed according to the participants' original treatment allocation in line with intention-to-treat principles. Data analysis on the continuous outcome measures will be performed with mixed-effect regression analyses for repeated measures with maximum likelihood estimation, with the assumption that missing data, if there will be any, are missing at random. The model will include fixed effects of time, treatment group, site and an interaction effect of treatment group by time, as well as random intercept and random slope to account for individual differences. A model with an interaction effect of treatment group, time and source of referral will be implemented to evaluate if source of referral is a moderator of treatment effect. Ordinal data will be analyzed with ordinal regression, binary data will be analyzed with logistic regression, and paired binary data will be analyzed with McNemar's tests. Logistic regression will be used to test if the blinded assessor´s guesses on treatment allocation are better than chance. Within- and between-group effect sizes will be reported as Cohen's d. We will carry out sensitivity analyses to test whether the results are robust enough even with missing data, as well as check if participants that break protocol influence the results. Additional analysis of the 1-year, 2-year and 5-year follow-ups data will evaluate if the treatment gains are maintained long-term.

Cost-effectiveness analysis

In our cost-effectiveness analysis, we will compare costs and health outcomes between stepped care ICBT and gold standard CBT. Costs will be collected from two different perspectives; a health care provider perspective, and a societal perspective. Costs within the health care provider perspective include recorded therapist time in the ICBT treatment and on face-to-face CBT sessions (including time for preparation and traveling), telephone calls, and administration. In the analysis from a societal perspective, other health care costs (other health care utilization and medication use) as well as indirect costs (e.g. informal care, productivity loss associated with school and work absenteeism) captured by the TiC-P will be included.[42] Our hypothesis is that there will be a difference between the two groups in treatment costs from the health care provider perspective, but we expect no difference in other costs.

Two types of analyses will be conducted on each of the two perspectives; a cost-effectiveness analysis, and a cost-utility analysis. In the cost-effectiveness analysis, both number of responders and remitters at primary endpoint (6-month follow-up) will be used as the health outcome. We will use expert consensus criteria to define treatment response and remission.[49] A responder is defined as at least 35% reduction on the CY-BOCS and a CGI-I score of 1 = "very much improved" or 2 = "much improved". Remission is defined as a score

of ≤12 on the CY-BOCS and a CGI-S rating of 1 = “normal, not at all ill” or 2 = “borderline mentally ill”. In the cost-utility analysis, the outcome will be the quality-adjusted life-years (QALYs), according to international standards for cost-effectiveness analyses.[50 51] QALYs will be calculated using the area under the curve method.[52] Costs will be estimated using linear regression analyses with 5000 bootstraps, as the distribution of costs is expected to be skewed towards lower values. As a global cost-effectiveness estimate, we will present the incremental cost-effectiveness ratio (difference in costs between the two groups divided by the difference in effect),[53] as well as a visual presentation of cost-effectiveness planes. The timeframe for the cost-effectiveness analyses will be from baseline to the 6-month follow-up.

TIME PLAN

2016 – spring 2017: Development of study infrastructure and routines, training of therapists and assessors.

Autumn 2017 – autumn 2019: Inclusion and treatment of participants, approx. 75 patients per year (50 patients in Stockholm and 25 patients in Gothenburg).

Autumn 2020: 6-month follow-up of last patients (primary endpoint), initiate data analysis and first study results

Winter 2020: 1-year follow-up of last patients

Winter 2021: 2-year follow-up of last patients

Winter 2024: 5-year follow-up of last patients

ETHICAL CONSIDERATIONS

A thorough assessment decreases the risk of patients in need of more extensive care being included in the study and these patients will be provided with proper referral information. For the ICBT patients, regular e-mail communication, and the possibility for the participating families to contact their therapist if needed, ensures that families with problems too severe for this program will be referred to more suitable treatments. The participants may worry about computer safety and maintenance of confidentiality. The families are provided with information about the risks and precautions that are being taken when using communication technology, e.g. encrypted server technology and double authentication with sms-logins. International rules and regulations will be followed. We have had no significant problems during our trials with paediatric OCD.

Adverse events will be assessed at post-treatment to capture unintended treatment effects. In case a participant (regardless of treatment condition) is getting worse during the treatment, an assessment will be conducted by the clinician and discussed with the PI to make necessary actions. There will be routines for clinicians about how to handle risk situations, and all participants will be monitors throughout the treatment to ensure their safety.

One strength in this study is that all participants get evidence-based treatment for OCD. The only difference is the format in which the treatment is delivered. This as opposition to other trials where one group is on the waitlist or receive a suboptimal treatment alternative as a comparison. Also, the non-responders will receive additional treatments after three months, which provide them with high-qualitative care during the follow-up period.

IMPLICATIONS

Building on our previous work, the current randomized non-inferiority trial will evaluate if ICBT can be delivered in a stepped care fashion without sacrificing efficacy and thus resulting in substantial resource savings. If, as predicted, the proposed stepped care model is shown to be non-inferior to and more cost-effective than gold standard treatment, we will aim to implement it in our specialist clinic within the next 5 years.

The trial will be organized within the clinical units BUP OCD and BUP CPF (Stockholm County Council) and BUP Specialmottagning (Region Västra Götaland).

The overall goal of our research is to develop a new format of effective treatment for paediatric OCD to help meet the high demand for specialist treatment and the shortage of

trained therapists to provide evidence based treatment for this prevalent condition. Given the increasing costs for the counties in Sweden[54] it is imperative to find new treatments that can provide effective and safe health care to a relatively low cost. This study has the potential to increase the availability of evidence-based treatment for young people with OCD, reducing geographical and other barriers, such as stigma. This would be desirable not only for patients and their families, but also for health care providers, who could potentially decrease their waiting times and costs.

References

1. American Psychiatric Association. Diagnostic and statistical manual of mental disorders : DSM-5. Washington, D.C: American Psychiatric Association 2013.
2. Valderhaug R, Ivarsson T. Functional impairment in clinical samples of Norwegian and Swedish children and adolescents with obsessive-compulsive disorder. *Eur Child Adolesc Psychiatry* 2005;14(3):164-73. doi: 10.1007/s00787-005-0456-9
3. Wewetzer C, Jans T, Muller B, et al. Long-term outcome and prognosis of obsessive-compulsive disorder with onset in childhood or adolescence. *Eur Child Adolesc Psychiatry* 2001;10(1):37-46.
4. McGuire JF, Piacentini J, Lewin AB, et al. A Meta-Analysis of Cognitive Behavior Therapy and Medication for Child Obsessive-Compulsive Disorder: Moderators of Treatment Efficacy, Response, and Remission. *Depress Anxiety* 2015;32(8):580-93. doi: 10.1002/da.22389
5. Hollander E, Kwon JH, Stein DJ, et al. Obsessive-compulsive and spectrum disorders: overview and quality of life issues. *J Clin Psychiatry* 1996;57 Suppl 8:3-6.
6. Mataix-Cols D, Marks IM. Self-help with minimal therapist contact for obsessive-compulsive disorder: a review. *Eur Psychiatry* 2006;21(2):75-80. doi: 10.1016/j.eurpsy.2005.07.003 [published Online First: 2005/12/20]
7. Shapiro DA, Cavanagh K, Lomas H. Geographic inequity in the availability of cognitive behavioural therapy in England and Wales. *Behav Cogn Psychother* 2003;31(02):185-92. doi: doi:10.1017/S1352465803002066
8. Griffiths F, Lindenmeyer A, Powell J, et al. Why are health care interventions delivered over the internet? A systematic review of the published literature. *J Med Internet Res* 2006;8(2):e10. doi: v8i2e10 [pii]
10.2196/jmir.8.2.e10 [published Online First: 2006/07/27]
9. Wootton BM, Titov N, Dear BF, et al. The acceptability of Internet-based treatment and characteristics of an adult sample with obsessive compulsive disorder: an Internet survey. *PLoS One* 2011;6(6):e20548. doi: 10.1371/journal.pone.0020548
10. Marques L, LeBlanc NJ, Weingarden HM, et al. Barriers to treatment and service utilization in an internet sample of individuals with obsessive-compulsive symptoms. *Depress Anxiety* 2010;27(5):470-5. doi: 10.1002/da.20694
11. Hedman E, Ljotsson B, Lindefors N. Cognitive behavior therapy via the Internet: a systematic review of applications, clinical efficacy and cost-effectiveness. *Expert Rev Pharmacoecon Outcomes Res* 2012;12(6):745-64. doi: 10.1586/erp.12.67
12. Abramowitz JS, Taylor S, McKay D. Obsessive-compulsive disorder. *Lancet* 2009;374(9688):491-9. doi: 10.1016/S0140-6736(09)60240-3
13. Andersson E, Enander J, Andren P, et al. Internet-based cognitive behaviour therapy for obsessive-compulsive disorder: a randomized controlled trial. *Psychol Med* 2012;42(10):2193-203. doi: 10.1017/S0033291712000244
14. Andersson E, Hedman E, Enander J, et al. D-Cycloserine vs placebo as adjunct to cognitive behavioral therapy for obsessive-compulsive disorder and interaction with antidepressants: a randomized clinical trial. *JAMA Psychiatry* 2015;72(7):659-67. doi: 10.1001/jamapsychiatry.2015.0546
15. Hauschildt M, Schröder J, Moritz S. Randomized-controlled trial on a novel (meta-) cognitive self-help approach for obsessive-compulsive disorder (“myMCT”). *Journal of Obsessive-Compulsive and Related Disorders* 2016;10:26-34.
16. Herbst N, Voderholzer U, Thiel N, et al. No talking, just writing! Efficacy of an internet-based cognitive behavioral therapy with exposure and response

prevention in obsessive compulsive disorder. *Psychother Psychosom* 2014;83(3):165-75.
17. Mahoney AE, Mackenzie A, Williams AD, et al. Internet cognitive behavioural treatment for obsessive compulsive disorder: A randomised controlled trial. *Behav Res Ther* 2014;63:99-106.
18. Wootton BM, Dear BF, Johnston L, et al. Remote treatment of obsessive-compulsive disorder: a randomized controlled trial. *Journal of Obsessive-Compulsive and Related Disorders* 2013;2(4):375-84.
19. Andersson E, Steneby S, Karlsson K, et al. Long-term efficacy of Internet-based cognitive behavior therapy for obsessive–compulsive disorder with or without booster: a randomized controlled trial. *Psychol Med* 2014;44(13):2877-87. doi: 10.1017/S0033291714000543
20. Wootton BM, Dear BF, Johnston L, et al. Self-guided internet-delivered cognitive behavior therapy (iCBT) for obsessive–compulsive disorder: 12 month follow-up. *Internet Interventions* 2015;2(3):243-47.
21. Andersson E, Hedman E, Ljótsson B, et al. Cost-effectiveness of internet-based cognitive behavior therapy for obsessive-compulsive disorder: results from a randomized controlled trial. *Journal of Obsessive-Compulsive and Related Disorders* 2015;4(0):47-53. doi: http://dx.doi.org/10.1016/j.jocrd.2014.12.004
22. Vigerland S, Ljotsson B, Thulin U, et al. Internet-delivered cognitive behavioural therapy for children with anxiety disorders: A randomised controlled trial. *Behav Res Ther* 2016;76:47-56. doi: 10.1016/j.brat.2015.11.006
23. Vigerland S, Thulin U, Ljótsson B, et al. Internet-delivered CBT for children with specific phobia: a pilot study. *Cogn Behav Ther* 2013;42(4):303-14.
24. Lenhard F, Andersson E, Mataix-Cols D, et al. Therapist-guided, internet-delivered cognitive-behavioral therapy for adolescents with obsessive-compulsive disorder: a randomized controlled trial. *J Am Acad Child Adolesc Psychiatry* 2017;56(1):10-19. doi: 10.1016/j.jaac.2016.09.515
25. Lenhard F, Vigerland S, Andersson E, et al. Internet-delivered cognitive behavior therapy for adolescents with obsessive-compulsive disorder: an open trial. *PLoS One* 2014;9(6):e100773. doi: 10.1371/journal.pone.0100773
26. Bonnert M, Ljótsson B, Hedman E, et al. Internet-delivered cognitive behavior therapy for adolescents with functional gastrointestinal disorders—an open trial. *Internet Interventions* 2014;1(3):141-48.
27. Bonnert M, Olén O, Lalouni M, et al. Internet-Delivered Cognitive Behavior Therapy for Adolescents With Irritable Bowel Syndrome: A Randomized Controlled Trial. *The American Journal of Gastroenterology* 2017;112(1):152-62.
28. Aspvall K, Andrén P, Lenhard F, et al. Internet-delivered cognitive behavioural therapy for young children with
Obsessive-Compulsive Disorder: Development and initial evaluation of the BIP OCD Junior programme. *Manuscript in preparation* 2017
29. Turner CM, Mataix-Cols D, Lovell K, et al. Telephone cognitive-behavioral therapy for adolescents with obsessive-compulsive disorder: a randomized controlled non-inferiority trial. *J Am Acad Child Adolesc Psychiatry* 2014;53(12):1298-307 e2. doi: 10.1016/j.jaac.2014.09.012
30. American Psychiatric Association. Diagnostic and statistical manual of mental disorders: DSM-5. 2013
31. Scahill L, Riddle MA, McSwiggin-Hardin M, et al. Children's Yale-Brown obsessive compulsive scale: reliability and validity. *J Am Acad Child Adolesc Psychiatry* 1997;36(6):844-52. doi: 10.1097/00004583-199706000-00023
32. Guy W. ECDEU Assessment Manual for Psychopharmacology. Rockville, Md: U.S. Department of Health, Education and Welfare 1976.
33. Shaffer D, Gould MS, Brasic J, et al. A children's global assessment scale (CGAS). *Arch Gen Psychiatry* 1983;40(11):1228-31.

34. Sheehan DV, Lecrubier Y, Sheehan KH, et al. The Mini-International Neuropsychiatric Interview (MINI): the development and validation of a structured diagnostic psychiatric interview for DSM-IV and ICD-10. *J Clin Psychiatry* 1998
35. Simpson HB, Maher M, Page JR, et al. Development of a Patient Adherence Scale for Exposure and Response Prevention Therapy. *Behav Ther* 2010;41(1):30-37. doi: http://doi.org/10.1016/j.beth.2008.12.002
36. Foa EB, Coles M, Huppert JD, et al. Development and validation of a child version of the obsessive compulsive inventory. *Behav Ther* 2010;41(1):121-32. doi: 10.1016/j.beth.2009.02.001
37. Stevens K. Assessing the performance of a new generic measure of health-related quality of life for children and refining it for use in health state valuation. *Applied Health Economics and Health Policy* 2011;9(3):157-69. doi: 10.2165/11587350-000000000-00000
38. Bastien CH, Vallieres A, Morin CM. Validation of the Insomnia Severity Index as an outcome measure for insomnia research. *Sleep Med* 2001;2(4):297-307. [published Online First: 2001/07/05]
39. Shafran R, Frampton I, Heyman I, et al. The preliminary development of a new self-report measure for OCD in young people. *J Adolesc* 2003;26(1):137-42. doi: 10.1016/S0140-1971(02)00083-0
40. Pinto A, Van Noppen B, Calvocoressi L. Development and preliminary psychometric evaluation of a self-rated version of the Family Accommodation Scale for Obsessive-Compulsive Disorder. *Journal of Obsessive-Compulsive and Related Disorders* 2013;2(4):457-65. doi: 10.1016/j.jocrd.2012.06.001
41. Allison C, Auyeung B, Baron-Cohen S. Toward Brief “Red Flags” for Autism Screening: The Short Autism Spectrum Quotient and the Short Quantitative Checklist in 1,000 Cases and 3,000 Controls. *J Am Acad Child Adolesc Psychiatry* 2012;51(2):202-12.e7. doi: 10.1016/j.jaac.2011.11.003
42. Bouwmans C, De Jong K, Timman R, et al. Feasibility, reliability and validity of a questionnaire on healthcare consumption and productivity loss in patients with a psychiatric disorder (TiC-P). *BMC Health Serv Res* 2013;13(1):217.
43. Angold A, Costello EJ, Messer SC, et al. Development of a short questionnaire for use in epidemiological studies of depression in children and adolescents. *Int J Methods Psychiatr Res* 1995;5:237-49.
44. Mataix-Cols D, Cowley AJ, Hankins M, et al. Reliability and validity of the Work and Social Adjustment Scale in phobic disorders. *Compr Psychiatry* 2005;46(3):223-28.
45. Mundt JC, Marks IM, Shear MK, et al. The Work and Social Adjustment Scale: a simple measure of impairment in functioning. *The British Journal of Psychiatry* 2002;180(5):461-64.
46. Borkovec TD, Nau SD. Credibility of analogue therapy rationales. *J Behav Ther Exp Psychiatry* 1972;3(4):257-60.
47. Andrusyna TP, Tang TZ, DeRubeis RJ, et al. The factor structure of the Working Alliance Inventory in cognitive-behavioral therapy. *The Journal of psychotherapy practice and research* 2001;10(3):173.
48. Attkisson CC, Zwick R. The Client Satisfaction Questionnaire: Psychometric properties and correlations with service utilization and psychotherapy outcome. *Eval Program Plann* 1982;5(3):233-37.
49. Mataix-Cols D, de la Cruz LF, Nordsletten AE, et al. Towards an international expert consensus for defining treatment response, remission, recovery and relapse in obsessive-compulsive disorder. *World psychiatry : official journal of the World Psychiatric Association* 2016;15(1):80-1. doi: 10.1002/wps.20299
50. Bridges JFP, Hauber AB, Marshall D, et al. Conjoint Analysis Applications in Health—a Checklist: A Report of the ISPOR Good Research Practices for

Conjoint Analysis Task Force. *Value Health* 2011;14(4):403-13. doi: 10.1016/j.jval.2010.11.013
51. Statens beredning för medicinsk och social utvärdering (SBU). Hälsoekonomiska utvärderingar [Internet]: SBU, 2017.
52. Matthews JNS, Altman DG, Campbell MJ, et al. Analysis Of Serial Measurements In Medical Research. *BMJ: British Medical Journal* 1990;300(6719):230-35.
53. Drummond MF, Sculpher MJ, Claxton K, et al. Methods for the economic evaluation of health care programmes. 4th ed. ed: Oxford, England : Oxford University Press 2015.
54. SKL. Ekonomirapporten oktober: SKL; 2015 [2016.
